# Localization of *Wolbachia*-like gene transcripts and peptides in adult *Onchocerca flexuosa* worms indicates tissue specific expression

**DOI:** 10.1186/1756-3305-6-2

**Published:** 2013-01-02

**Authors:** Samantha N McNulty, Kerstin Fischer, Kurt C Curtis, Gary J Weil, Norbert W Brattig, Peter U Fischer

**Affiliations:** 1 Infectious Diseases Division, Department of Internal Medicine, Washington University School of Medicine, Saint Louis, MO, 63110, USA; 2 Bernhard Nocht Institute for Tropical Medicine, Hamburg, Germany

**Keywords:** Filariasis, horizontal gene transfer, *in situ* hybridization, immunohistology, *Wolbachia*

## Abstract

**Background:**

Most filarial species in the genus *Onchocerca* depend on *Wolbachia* endobacteria to successfully carry out their life cycle. *O. flexuosa* is a *Wolbachia*-free species, but its genome contains *Wolbachia*-like sequences presumably obtained from *Wolbachia* via horizontal gene transfer. Proteogenomic studies have shown that many of these *Wolbachia*-like sequences are expressed in adult worms.

**Methods:**

Six *Wolbachia*-like sequences in *O. flexuosa* were chosen for further study based on their sequence conservation with *Wolbachia* genes, length of predicted open reading frames, and expression at the RNA and/or protein levels. *In situ* hybridization and immunohistochemical labeling were used to localize *Wolbachia*-like transcripts and peptides in adult worm tissues.

**Results:**

RNA probes representing three of the six target sequences produced hybridization signals in worm tissues. These probes bound to transcripts in the intestine and lateral chords of both sexes, in the hypodermis, median chords and uteri in females, and in sperm precursor cells in males. Antibodies raised to three peptides corresponding to these transcripts bound to specific bands in a soluble extract of adult *O. flexuosa* by Western blot that were not labeled by control antibodies in pre-immune serum. Two of the three antibodies produced labeling patterns in adult worm sections that were similar to those of the RNA probes, while the third produced a different pattern.

**Conclusions:**

A subset of the *Wolbachia*-like sequences present in the genome of the *Wolbachia*-free filarial species *O. flexuosa* are transcribed in tissues where *Wolbachia* reside in infected filarial species. Some of the peptides and/or proteins derived from these transcripts appear to be concentrated in the same tissues while others may be exported to other regions of the worm. These results suggest that horizontally transferred *Wolbachia* genes and gene products may replicate important *Wolbachia* functions in uninfected filarial worms.

## Background

Filarial nematodes comprise a superfamily of parasitic worms that infect a wide array of hosts, including humans. Three filarial species (*Onchocerca volvulus, Wuchereria bancrofti*, and *Brugia malayi*) are important human pathogens in the tropics, and many other filarial species infect wild or domestic animals throughout the world. One feature shared by many filarial pathogens is their association with *Wolbachia* endobacteria. Prior studies have shown that *Wolbachia* play important roles in worm growth, molting, reproduction, and pathogenesis [1-4].

Filarial nematodes have been divided into eight subfamilies based on classical parasitological criteria. Two of these subfamilies, the Onchocercinae and Dirofilariinae, appear to be dominated by *Wolbachia*-dependent species [5-7]. The abundance of *Wolbachia*-dependent species relative to *Wolbachia*-free species and the congruencies between the phylogenetic lineages of *Wolbachia* and their filarial hosts suggest that *Wolbachia* entered the filarial lineage prior to the differentiation of the Onchocercinae and Dirofilariinae [5-7]. Recent studies have shown that two *Wolbachia*-free filarial species, *Onchocerca flexuosa* (subfamily: Onchocercinae) and *Acanthocheilonema viteae* (subfamily: Dirofilariinae), contain *Wolbachia*-like DNA sequences in their nuclear genomes, indicating that these species may have been associated with *Wolbachia* in the ancient past [8]. Since *O. flexuosa* and *A. viteae* are relatively distantly related [6,9], we expect that this trend will prove consistent among other *Wolbachia*-free onchocercids and dirofilariids.

Our previous surveys of the genome and transcriptome of *O. flexuosa* identified sequence fragments with similarity to 178 different *Wolbachia* genes [8,10]. qRT-PCR reactions and partial transcriptome sequencing indicated that many of these *Wolbachia*-like sequences are transcribed, and a mass spectrometry study with follow-up immunohistology and Western blot studies indicated that at least two *Wolbachia*-like sequences were translated into *Wolbachia*-like peptides [10]. *Wolbachia* are known to be proficient at transferring genetic material to host cells and many *Wolbachia*-like sequences are present in the genomes of *Wolbachia*-dependent filarial worms; however, the *Wolbachia*-like sequences found in the genomes of dependent species like *B. malayi* are thought to be degenerate and non-functional [11]. Retention and expression of *Wolbachia*-like sequences in *O. flexuosa* suggests that they may have essential roles in the biology of *Wolbachia*-free filarial worms.

Prior work has shown that *Wolbachia* endobacteria are restricted to specific tissues in filarial nematodes [7,12-16]. In early development, the vertically transmitted bacteria that were present in the fertilized oocyte segregate to cells that give rise to the hypodermal lateral chords [13]. *Wolbachia* from the lateral chords then invade the ovaries and testis prior to sexual maturation [12,17]. This pattern of localization (i.e., lateral chords and reproductive tissues) may be critical to *Wolbachia*’s function as a mutualist and to its role in development and reproduction. Therefore, localization studies were performed to gain insight into the possible function(s) of transferred and retained *Wolbachia*-like sequences in the *Wolbachia-*free filarial species *O. flexuosa*.

The present study used *in situ* hybridization to localize expression of *Wolbachia*-like transcripts in adult *O. flexuosa* and immunohistochemical staining to localize peptides corresponding to these sequences. Thus far, all of the RNA probes that produce a signal in adult tissues stain the intestine and lateral chords in both sexes, the hypodermis, median chords, and uteri of females, and developing sperm in males. Two of the three *Wolbachia*-like peptides were identified in the same tissues and in developing embryos within the females. The third *Wolbachia*-like peptide was found in similar tissues and in the basal areas of somatic muscles.

## Methods

### Parasite material

*O. flexuosa* nodules were cut from the skins of freshly-shot European red deer (*Cervus elaphus*) following community hunts in northern Germany (Schleswig-Holstein) as previously described [18]. Adult *B. malayi* were obtained from the Filariasis Research Reagent Resource Center [19]. Worms to be used for RNA or DNA isolation were stored in TRIzol (Invitrogen, Carlsbad, CA, USA) or 1x phosphate buffered saline, respectively, at -80°C. *O. flexuosa* nodules intended for histological examination were fixed in 4% buffered formalin, embedded in paraffin and sectioned at 5μm thickness according to standard histological procedure.

### Nucleic acid isolation

DNA was isolated using an E.Z.N.A Tissue DNA Kit (Omega Bio-Tek, Norcross, GA, USA) according to the manufacturer’s suggested protocol. Total RNA was isolated by homogenization in TRIzol (Invitrogen) and organic extraction with 1-bromo-3-chloropropane and purified using an RNeasy Mini Kit with the optional on-column DNase digest (Qiagen, Valencia, CA, USA). A second DNase treatment was performed using the Turbo DNA-free kit (Invitrogen). cDNA was synthesized using qScript cDNA SuperMix (Quanta, Gaithersburg, MD, USA) and tested for DNA contamination by PCR with intron-spanning primer sets as previously described [8].

### Bioinformatic analyses

The *O. flexuosa* adult transcriptome was sequenced using Roche/454 Titanium technology and assembled using the Newber v2.5 assembler as described previously [10]. Sequences are available from the Genbank transcriptome shotgun assembly database (BioProject number 62565, accession numbers JI459010-JI484230) and from Nematode.net [20]. *Wolbachia*-like sequences were identified from *O. flexuosa* transcripts by BLASTX search against the non-redundant protein database [10]. Measurements of sequence conservation were based on the e-values of hits obtained in this blast search. The relative expression of each *O. flexuosa* isogroup (unique sequence locus) was estimated based on the number of reads included in each assembled isogroup normalized to the total length of the isogroup (the summed length of all contigs included in the isogroup) (Additional file 1: Table S1). Putative *Wolbachia*-like peptides were identified by blasting six-frame translations of *O. flexuosa* transcripts against the non-redundant protein database (>50% sequence identity shared with *Wolbachia* protein, bit score >35).

### RNA probe construction and *in situ* hybridization

Probes were constructed as previously described [21]. Briefly, 300-600 bp target sequences were PCR amplified from cDNA (see Additional file 2: Table S2 for primers) and cloned using the TOPO TA Cloning Kit Dual Promoter (Invitrogen). Plasmids were purified with the QIAprep Spin Miniprep Kit (Qiagen) and linearized by restriction digest with either EcoRV or BamHI. Following purification of linearized plasmid (QIAquick PCR Purification Kit, Qiagen), biotinlyated antisense RNA probes and sense controls were transcribed from the plasmid template using the MEGAscript SP6 and T7 in vitro transcription kits (Invitrogen) with biotinylated NTPs (Roche Diagnostics, Indianapolis, IN, USA). The final biotinlyated RNA probes were purified and concentrated by ethanol precipitation and stored in 1 × tris-EDTA buffer, pH 8.0.

Slides from various nodules were tested with control probes to determine the quality of RNA preservation. This step is necessary due to the short lifespan of *O. flexuosa*, as studies have indicated that 20% of nodules collected (even from very young deer) contain dead worms [18]. *In situ* hybridizations showed inconsistent results in older, mf producing worms. This may have been due to reduced penetration of the fixative through larger, tougher nodules or due to reduced transcription rates in the older worms. Slides prepared from small, soft nodules containing young adult worms showed better RNA integrity. Therefore, we chose to focus on young worms in our *in situ* hybridization studies.

*In situ* hybridizations using formalin-fixed, paraffin-embedded specimens were carried out as previously described [12]. Briefly, slides were hybridized with 1μg/mL of RNA probe in hybridization buffer (50% formamide, 5x SSC, 0.3mg/mL yeast tRNA, 100μg/mL heparin, 1x Denhardt’s reagent, 0.1% CHAPS and 5mM EDTA) overnight at 60°C. Stringency washes (60°C for 30m) were carried out using reagents from the “*In situ* Hybridization Detection System” (Dako, Carpinteria, CA, USA). The same kit was used for colorimetric detection according to the manufacturer’s suggested protocol. Slides were viewed using an Olympus-BX40 microscope (Olympus, Tokyo, Japan) and photographed with an Infinity2 digital microscope camera using Infinity Capture software (Lumenera, Ottowa, Ontario, Canada). For fluorescent detection, washed sections were incubated with 5μg/mL streptavidin Alexa Fluor 488 (Invitrogen) in 1x phosphate buffered saline with 0.1% bovine serum albumin in the dark, at room temperature. After 20 minutes, 5μg/mL wheat germ aggutinin Alexa Fluor 633 was applied to the slide to highlight cell membranes, and the incubation was allowed to proceed for 10 more minutes. Finally, sections were rinsed in 1x tris buffered saline and cover slips were mounted with ProLong Gold Antifade Reagent with DAPI (Invitrogen). Fluorescent labeling was viewed with a Zeiss Axioskop 2 Mot Plus fluorescence microscope equipped with an Axiocam MRm monochrome camera, and images were captured using Axiovision 4.6 software (Carl Zeiss Inc., Thornwood, NY, USA).

### Antibody production

Anti-peptide antibodies were produced and purified by LifeTein LLC (South Plainfield, NJ, USA). Targeted portions of *Wolbachia*-like peptides were selected based on predicted chemical and immunogenic properties (Additional file 3: Table S3). Target peptides were synthesized and coupled to a keyhole limpit hemocyanin (KLH) carrier protein. Rabbits were bled to collect pre-immune serum and then immunized with the peptide/KLH conjugates. Antibody production was allowed to proceed for 12-15 weeks with 3 booster immunizations. Following the terminal bleed, polyclonal antibodies were affinity purified from serum using the target peptide (without the KLH carrier) and tested by ELISA prior to use. Polyclonal antibodies against the KLH carrier protein were produced and purified in the same manner. Total IgG was purified from rabbit pre-immune sera using the Protein A Agarose Kit (KPL, Gaithersburg, MD, USA).

### Binding of antibodies to *Wolbachia*-like peptides present in *O. flexuosa* antigen extract by Western blot

Western blots were performed as previously described [10]. Briefly, *O. flexuosa* total worm homogenate separated by SDS-PAGE gel electrophoresis and transferred to a nitrocellulose membrane. Blot strips were probed with peptide antibodies (2.5μg/mL for antibodies against the peptides from isotig12596 and isotig21532, 5μg/mL for antibodies against the HlyD peptide) and purified IgG from the corresponding rabbits’ pre-immune sera (5μg/mL from rabbits used to produce antibodies against the peptides from isotig12596 and isotig21532, 10μg/mL from the rabbit used to produce antibodies against the HlyD peptide) in 1x phosphate buffered saline with 0.5% Tween (PBS/T) overnight at 4°C and washed with PBS/T at room temperature. Anti-rabbit IgG(Fc) AP conjugate (Promega, Sunnyvale, CA, USA) was diluted 1:3,500 in PBS/T and applied to the strips for 1h at 37°C. Strips were again washed with PBS/T and developed using NBT/BCIP substrate (Promega).

### Immunohistochemical labeling

Various dilutions of primary antibodies were tested in order to optimize signal/background. Antibodies against peptides from isotig12596, isotig21532, HlyD and KLH (negative control) were used at 9.7μg/mL, 5.7μg/mL, 7.4μg/mL, and 1.4 μg/mL, respectively. Visualization was mostly performed with alkaline phosphatase anti-alkaline phosphatase reagents (Dako) as previously described [12,22], and slides were viewed using an Olympus-BX40 microscope (Olympus) and photographed with an Infinity2 digital microscope camera using Infinity Capture software (Lumenera). FITC labeled goat anti-mouse IgG (1:300; Sigma, St. Louis, MO, USA) was used as secondary antibodies for immunofluorescent labeling, and DAPI and wheat germ agglutinin Alexa Fluor 633 conjugate were used to visualize DNA and cell membranes, respectively (Invitrogen). Fluorescent labeling was viewed with a Zeiss Axioskop 2 Mot Plus fluorescence microscope equipped with an Axiocam MRm monochrome camera, and images were captured using Axiovision 4.6 software (Carl Zeiss Inc.).

## Results and discussion

### Target selection

**Figure 1:**
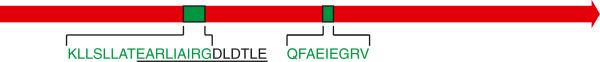
***Wolbachia*-like peptides identified by mass spectrometry analysis of *Onchocerca flexuosa *lysate.** In a recent proteomic analysis, two peptides detected in *O. flexuosa* adult worm lysate (shown in green) were mapped to an HlyD family secretion protein from the *Wolbachia* endosymbiont of *Culex quinquefasciatus*, accession number YP_001974912 [10]. Polyclonal antibodies were raised against one of these peptides. The detected peptide, in its entirety, was predicted to be unfavorable for antibody production. Our selected epitope contains half of the detected peptide extended by six amino acids to the C-terminus to enhance solubility and immunogenic properties (underlined).

Genomic and transcriptomic surveys have shown that at least 178 different *Wolbachia* genes are represented by sequence fragments in the genome of *O. flexuosa*[8,10]. In our previous mass specrometry analysis of *O. flexuosa* adult worm lysate, three unique peptides mapped to two *Wolbachia* proteins included in our comparative database [10]. One peptide mapped to a lipoprotein releasing system trans-membrane protein (LolC) present in several *Wolbachia* strains. The localization of this peptide and its corresponding RNA were previously described [10]. The other two peptides mapped to an HlyD family secretion protein from the *Wolbachia* endosymbiont of *Culex quinquefasciatus* (Figure 1). Like the LolC protein, HlyD is a transmembrane component of a bacterial ABC transport system [23]. Of the two peptides that mapped to HlyD, we chose to focus on the one predicted to have the most favorable chemical and immunogenic qualities for further analysis despite the fact that no corresponding sequence has been identified in among our (as yet incomplete) set of *O. flexuosa* genomic and transcriptomic sequences.

None of the *Wolbachia*-like peptides predicted from the *O. flexuosa* transcriptome were identified in our previous proteomic study [10]. While this could certainly be due to lack of expression, it is quite likely that the *Wolbachia*-like peptides are difficult to detect in total worm lysate due to their low abundance compared to other *O. flexuosa* proteins. Indeed, *Wolbachia* proteins have proven difficult to detect using this method even in *Wolbachia*-dependent species [24]. Therefore, a targeted approach employing specific anti-peptide antibodies was used to search for evidence of protein-level expression among our *Wolbachia*-like transcript sequences. We decided on three potential indicators of importance to determine which of the *Wolbachia*-like sequences warranted further examination: sequence conservation with *Wolbachia*, putative size of the open reading frame, and expression level (Table 1). Purifying selection should protect sequences that are vital for the parasite. Therefore, we focused on sequences that were most similar to the presumed donor sequence from *Wolbachia* (or the closest proxy for the donor sequence included in public databases) and sequences with long open reading frames (i.e., without numerous stop codons and frameshift mutations). Though no exact criteria for biologically relevant expression levels are defined, it is generally assumed that abundantly expressed genes are important. Some of the isogroups containing *Wolbachia*-like sequences are expressed at relatively low (presumably background) levels, while others are expressed to a much higher degree (Additional file 1: Table S1). The top ten most abundantly expressed *Wolbachia*-like sequences all had expression levels higher than the average and median expression seen among all *O. flexuosa* isogroups. Ultimately, five *Wolbachia*-like sequences fitting our criteria were selected for analysis (Table 2). These include sequences similar to GMP synthase, aminopeptidase P, succinyl-diaminopimelate desuccinylase, citrate synthase, and a *Wolbachia* hypothetical protein. According to the COG and KEGG databases [25-28], GMP synthase is involved in purine biosynthesis, aminopeptidase P is involved in amino acid transport and metabolism, succinly-diaminopimelate desuccinylase is involved in lysine biosynthesis, and citrate synthase is involved in the Krebs cycle. The function of the *Wolbachia* hypothetical protein is, of course, unknown.

**Table 1:** **Ranked lists of *Onchocerca flexuosa Wolbachia*-like transcripts with highest expression levels, highest sequence conservation with *Wolbachia *proteins, and longest open reading frames**

**Most abundantly expressed**	**Greatest sequence conservation with *Wolbachia* proteins**	**Longest open reading frames**
isogroup00138	isotig12596 (isogroup04608)	isotig21532 (isogroup13474)
isogroup01651	isotig12597 (isogroup04608)	isotig17946 (isogroup09888)
isogroup00994	isotig14150 (isogroup06092)	F2XBAMM02FMIGV (singleton)
isogroup09858	isotig12363 (isogroup04466)	isotig16102 (isogroup08044)
isogroup02611	isotig23404 (isogroup15346)	F2XBAMM02GZVCP (singleton)
isogroup04316	isotig17716 (isogroup09658)	F2XBAMM02JSI7M (singleton)
isogroup00988	isotig14485 (isogroup06427)	F2XBAMM02IC59R (singleton)
isogroup04702	GMRH4OU01D83JZ (singleton)	isotig14485 (isogroup06427)
isogroup02658	isotig18970 (isogroup10912)	isotig12596 (isogroup04608)
isogroup06261	F2XBAMM02GHDMV (singleton)	GMRH4OU02FRNRL (singleton)
isogroup04608	GMRH4OU02JV82A (singleton)	contig10287 (isogroup00994)
isogroup02939	isotig20687 (isogroup12629)	isotig21343 (isogroup13285)
isogroup04541	GMRH4OU02GI0KU (singleton)	isotig12363 (isogroup04466)
isogroup13474	F2XBAMM02F31KT (singleton)	isotig13398 (isogroup05340)
isogroup00429	F2XBAMM02G13WR (singleton)	isotig18970 (isogroup10912)

Expression was assessed based on the number of sequencing reads associated with each isogroup (see Methods), and sequence conservation was assessed based on the e-score of the blast hit to a *Wolbachia* protein in a BLASTX against the non-redundant protein database.

**Table 2:** ***Wolbachia*-like sequences from the *Onchocerca flexuosa *transcriptome chosen for localization studies**

**Isogroup**	**Isotig**	**Accession of best BLAST match**	***Wolbachia* strain of best BLAST match**	**Annotation of best BLAST match**	**Targeted epitope**
isogroup00994	contig10287	YP_198273	*Brugia malayi*	GMP synthase	KSHHNVGRLPKKMNLK
isogroup04608	isotig12596	ZP_03787836	*Muscidifurax uniraptor*	aminopeptidase P	DSGGQYLDGTTDLIR
isogroup13474	isotig21532	CAL29441	*Onchocerca volvulus*	hypothetical protein OW4-D	HRKHNQESKSEELFS
isogroup06427	isotig14485	NP_966542.1	*Drosophila melanogaster*	succinyl-diaminopimelate desuccinylase	SNRGAFFLTPDRSID
isogroup04466	isotig12363	YP_002727485	*Drosphila simulans*	citrate synthase	YEMMSDKETNGTLP

Foster et al. [29] reported that the *Wolbachia* endosymbionts of *B. malayi* are capable of de novo nucleotide synthesis, and they suggested that the bacteria may play a role in metabolic provisioning. A recent analysis of the genome of the *Wolbachia* endosymbiont of *Onchocerca ochengi* further supported this hypothesis, suggesting that *Wolbachia* may supplement the host nucleotide pool [30]. It is interesting that enzymes related to energy metabolism and purine synthesis were identified using our selection algorithm, which did not include potential enzymatic function as a selection criterion. Future work will be required to determine whether these sequences encode enzymes that are functional in *O. flexuosa*.

### *In situ* hybridization

**Figure 2:**
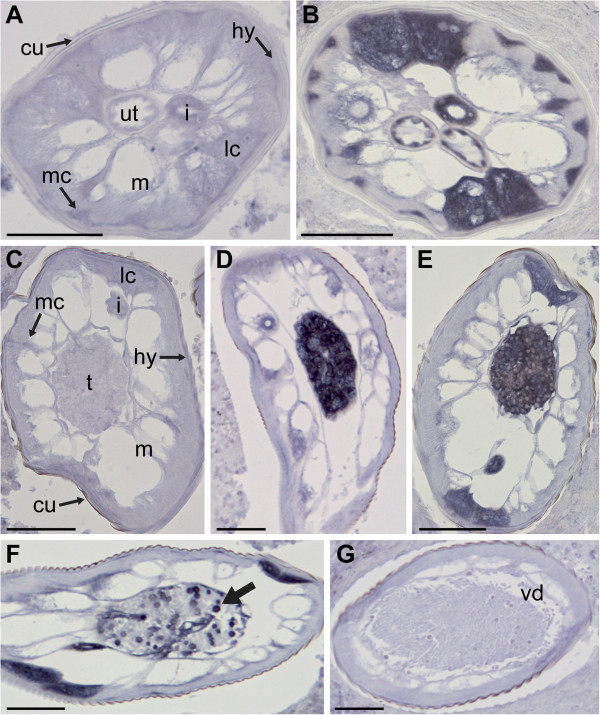
**Localization of a putative *Wolbachia*-like aminopeptidase P transcript in adult *Onchocerca flexuosa. *** While the sense RNA probe (negative control) did not label tissues in female (**A**) or male (**C**) worms, the antisense probe against isotig12956 (which has sequence similarity to an aminopeptidase P gene from the *Wolbachia* endosymbiont of *Muscidifurax uniraptor*) produced a strong signal in both sexes. The hypodermis, lateral chords, median chords, intestine, and the uterine epithelium were intensely labeled in young, non-reproductive females (**B**). The same probe labeled small, round spermatogonia (**D**) and larger spermatocytes (**E**) within the male testis. The large, round, secondary spermatocytes (arrow) were labeled in the transitional region between the testis and vas deferens, whereas the smaller spermatids were not (**F**). No labeling was observed in the elongated, mature sperm within the vas deferens (**G**). The labeling of the lateral chords was most intense in the male mid-body and faded towards the posterior end of the males in the vicinity of the mature sperm. Abbreviations: cu, cuticle; hy, hypodermis; m, muscle; lc, lateral chords; mc, median chords; i, intestine; ut, uterus; t, testis; vd, vas deferens. Scale bar = 50μm.

RNA probes corresponding to the five sequences of interest selected from the *O. flexuosa* transcriptome and *Wolbachia* HlyD were constructed and used in *in situ* hybridization studies. Because no genomic or transcriptomic sequence related to *Wolbachia* HlyD has been found in *O. flexuosa*, the HlyD probe was amplified from the *Wolbachia* endosymbiont of *B. malayi*. Probes against isotig12596 and *Wolbachia* HlyD produced strong signals in *O. flexuosa* tissues (Figure 2 and Additional file 4: Figure S1, respectively). The probe against *O. flexuosa* isotig21532 produced a much weaker signal (Additional file 5: Figure S2), and the probes against isotig14485, isotig12363 and contig10287 produced no signal at all. This was surprising, because expression levels for these transcripts were not estimated to be significantly lower than that of isotig12596 (Additional file 1: Table S1). It could be that these three sequences are expressed under conditions that are not represented in the particular worm specimens we examined or that some *Wolbachia*-like sequences are expressed at a low level throughout the body rather than concentrating in specific tissues, thus making them difficult to detect using this method. It is also possible that technical issues with the RNA probes led to this result.

To date, all of the RNA probes against *Wolbachia*-like sequences that have successfully labeled *O. flexuosa* tissues produced very similar labeling patterns (see [8,10], Figure 2, Additional file 4: Figure S1, and Additional file 5: Figure S2). The results of *in situ* hybridizations with the probe against isotig12596 (putative aminopeptidase P) are shown as an example (Figure 2). Intense labeling was seen in the lateral chords and intestine of both sexes (Figure 2B, E, and F), in the hypodermis, in the empty uteri of young females (Figure 2B), and in the testis and developing sperm of males (Figure 2D-F). Mature sperm in the vas deferens were not labeled, and the signal in the lateral chords decreased towards the tail end of the male worm where the mature sperm reside (Figure 2G).

**Figure 3:**
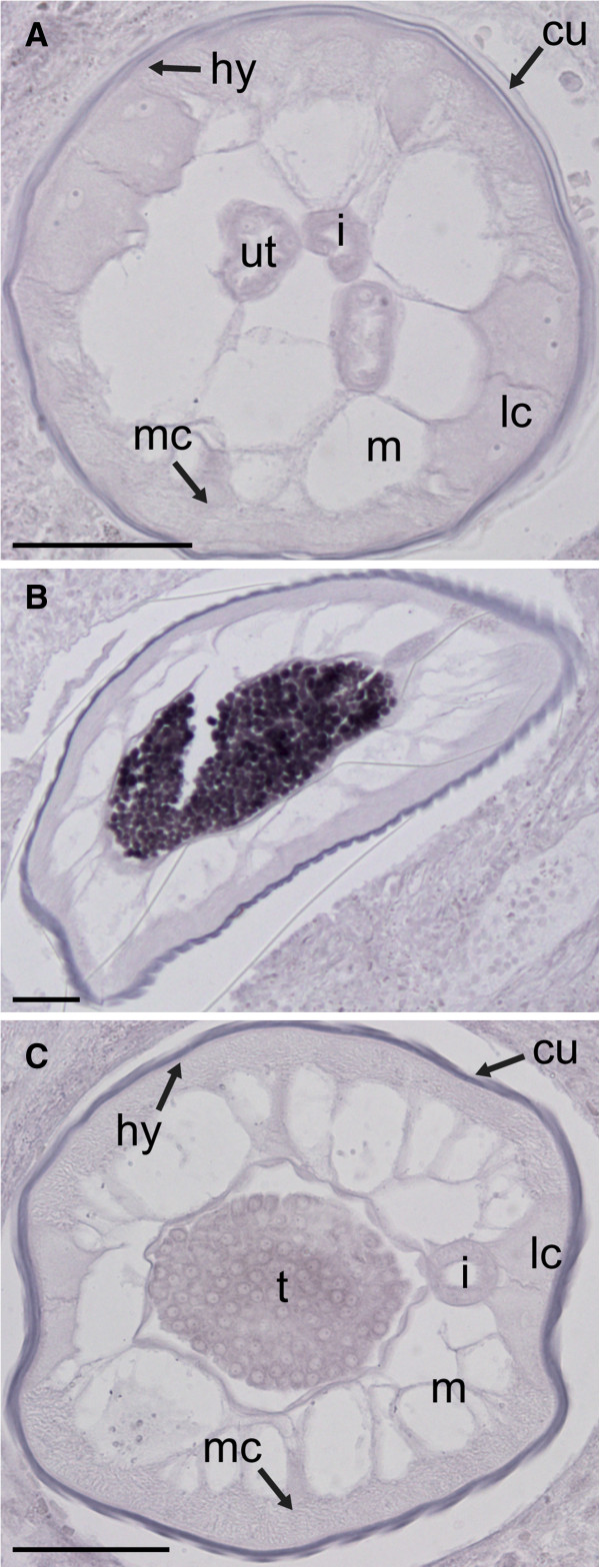
**Localization of the major sperm protein 1 transcript in *Onchocerca flexuosa. ***A probe against major sperm protein 1 of *B. malayi* (described by [21] was used as a control since it is unrelated to *Wolbachia* and has a well-characterized, tissue-specific expression pattern. No labeling was seen in female worms (**A**). Whereas the spermatocytes within the male testis show strong labeling (**B**), other stages do not (**C**). No staining is seen in the lateral chords in any region of the male worm. This result agrees with the localization pattern reported in *B. malayi*[21], and it is very different from the *in situ* hybridization patterns observed with probes against *Wolbachia*-like sequences. Abbreviations: cu, cuticle; hy, hypodermis; m, muscle; lc, lateral chords; mc, median chords; i, intestine; ut, uterus; t, testis. Scale bar = 50μm.

The intestine, lateral chords and the reproductive organs are among the most metabolically active tissues in the adult worm. Since these tissues could be responsible for the production of many gene products, we tested probes unrelated to the *Wolbachia*-like sequences to ensure that the detected pattern is specific to this subset of sequences. As expected, unrelated probes produced dissimilar labeling patterns, as was shown for a probe targeting transcripts derived from the major sperm protein gene (Figure 3) [21].

A shared expression pattern can be taken as evidence that a group of genes is under the control of either the same or functionally similar promoters or other regulatory elements. Approximately 15% of the genes encoded by *B. malayi* are organized into operons [31,32]. Since the complete genome of *O. flexuosa* has not been sequenced, we cannot rule out the possibility that some of the *Wolbachia*-like sequences are organized in this way, giving rise to a characteristic pattern of expression. However, clusters of *Wolbachia*-like sequences were not identified in genomic surveys of *O. flexuosa*[8], so it is unlikely that they are all organized into operons.

*Wolbachia* are commonly detected in the lateral chords, embryos, developing (but not mature) sperm, and sometimes in the intestine of infected filarial nematodes [7,12]. Interestingly, the expression pattern of *Wolbachia*-like sequences in *O. flexuosa* mimics the distribution of *Wolbachia* in infected species. This may indicate that some of the same tissues (e.g., lateral chords, reproductive organs, intestine etc.) that harbor *Wolbachia* in infected species produce *Wolbachia*-related products in *Wolbachia*-independent species.

### Western blot results

**Figure 4:**
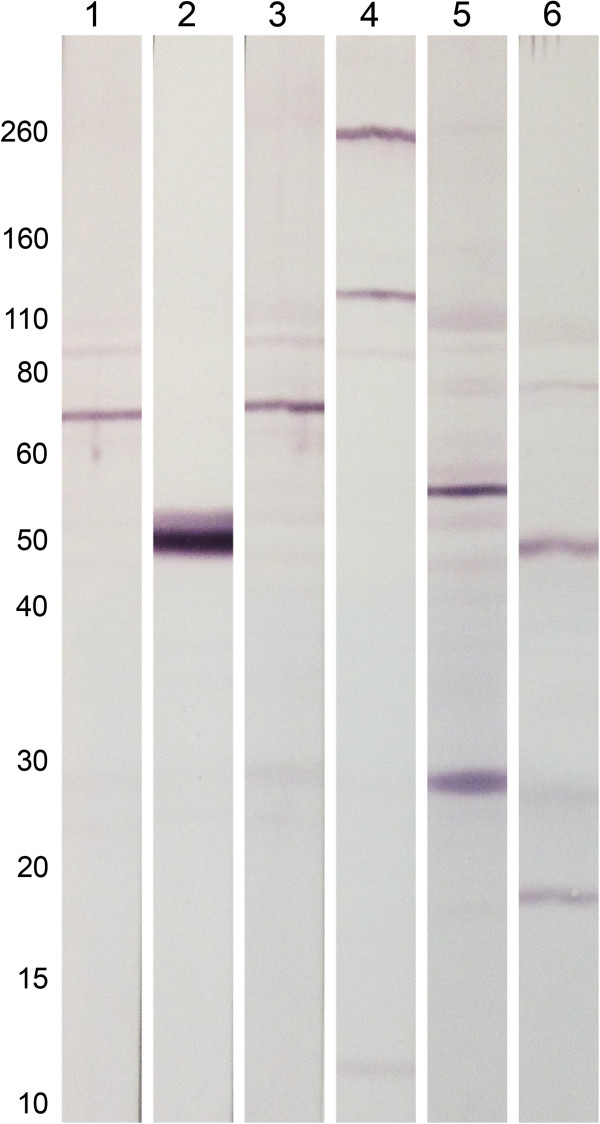
**Western blot detection of putative peptides with antibodies to peptides corresponding to isotig12596, isotig21532, and *Wolbachia *HlyD in *Onchocerca flexuosa *adult worm lysate.** Affinity-purified rabbit polyclonal antibodies against a peptide predicted from isotig12596 recognized a band at approximately 50kDa (lane 2). Antibodies raised against a peptide predicted from isotig21532 recognized two main bands at approximately 260 and 130 kDa (lane 4). Antibodies against a peptide from *Wolbachia* HlyD labeled two main bands at approximately 47 and 18 kDa (lane 6). Purified IgG from the pre-immune serum of the corresponding rabbits did not label these bands (lanes 1, 3 and 5). Approximate molecular masses (kDa) are shown on the left side of the blot.

Polyclonal antibodies were raised against a peptide from HlyD (Figure 1) and translated peptides from isotig12596, and isotig21532 (see Table 2). These were used to probe *O. flexuosa* lysate by Western blot (Figure 4). Antibodies to the predicted peptide from isotig12596 specifically bound to a protein band at approximately 50 kDa, while antibodies against the predicted peptide from isotigs21532 and the *Wolbachia* HlyD peptide each bound to two protein bands (molecular weights of approximately 120 and 260 kDa for the antibodies to the predicted peptide from isotig21532, and approximately 48 and 18 kDa for the antibodies to the HlyD peptide). Though the detection of multiple bands could be a sign of cross reactivity, it could also reflect multiple splice isoforms, post-translational modification, proteolytic processing, or degradation. IgG from the same rabbits’ pre-immune sera did not bind to bands recognized by their immune sera.

It is not possible to estimate the masses of the *O. flexuosa* proteins based on our transcriptome data due to heavy fragmentation. The masses of the top blastx matches to isotigs12596 (aminopeptidase P from the *Wolbachia* endosymbiont of *Muscidifurax uniraptor*) and 21532 (a hypothetical protein from the *Wolbachia* endosymbiont of *Onchocerca volvulus*) and the HlyD protein are estimated to be approximately 58 kDa, 83 kDa, and 56 kDa, respectively, based on their reported amino acid sequences. The band detected by the antibodies to the predicted peptide from isotig12596 was similar in size to the corresponding *Wolbachia* protein (50 kDa vs. 58 kDa), as was one of the bands detected by the antibodies to the HlyD peptide (48 kDa vs. 56 kDa). The bands detected by the antibodies to the predicted peptide from isotig21532 were much larger than the putative *Wolbachia* homolog (120 and 260 kDa vs. 83 kDa), perhaps due to the incorporation of extra domains from the *O. flexuosa* genome. Of course, the full amino acid sequences of the *O. flexuosa* proteins detected by these antibodies are not known at this time.

### Immunohistology

**Figure 5:**
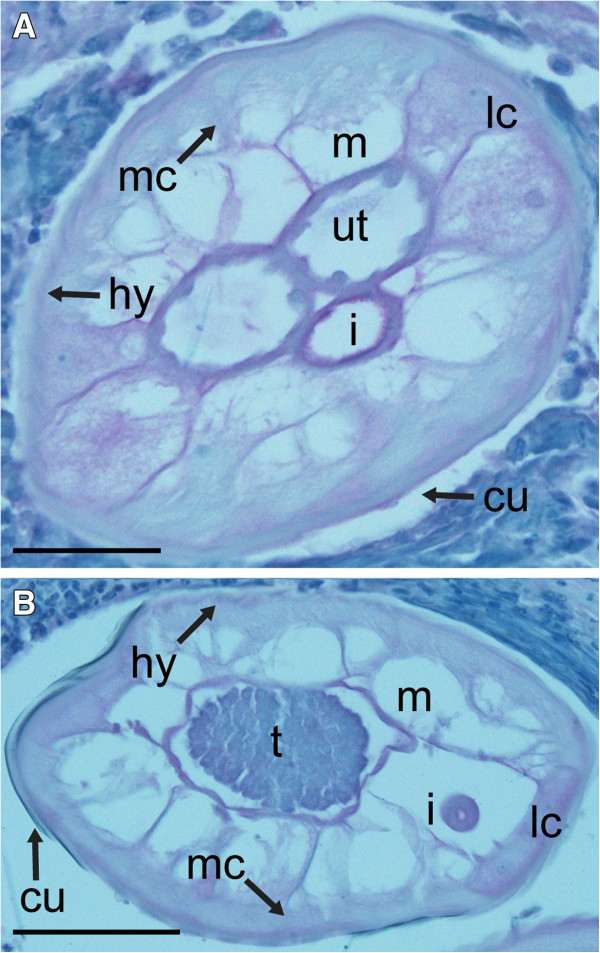
**Immunohistochemical labeling of adult *Onchocerca flexuosa *with polyclonal antibodies against keyhole limpet hemocyanin (KLH).** Keyhole limpit hemocyanin was used as a carrier protein to generate antibodies against putative *Wolbachia*-like peptides. Antibodies against this carrier failed to bind specific tissues in female (**A**) or male (**B**) worms. Abbreviations: cu, cuticle; hy, hypodermis; m, muscle; lc, lateral chords; mc, median chords; i, intestine; ut, uterus; t, testis. Scale bar = 50μm.

**Figure 6:**
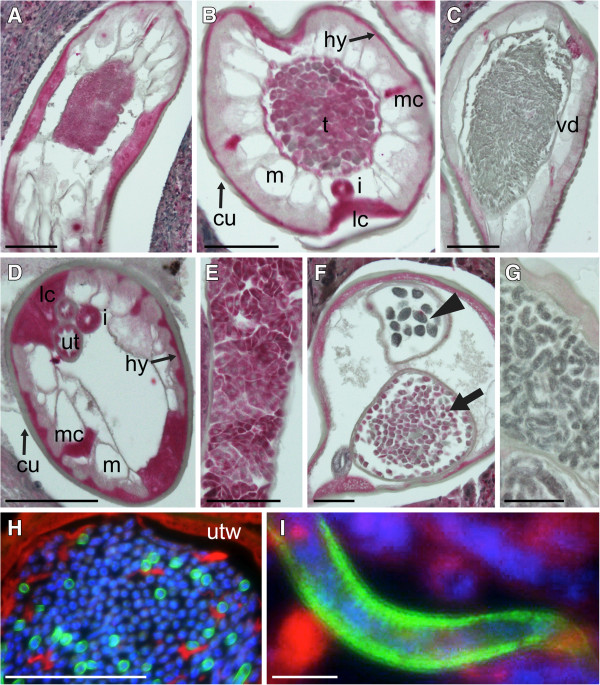
**Localization of the putative HlyD peptide in *Onchocerca flexuosa *by immunohistochemical labeling.** Polyclonal antibodies raised against a peptide from the HlyD protein of the *Wolbachia* endosymbiont of *Culex quinquefasciatus* labeled the intestine and hypodermis, lateral chords, and median chords in male worms (**A**, **B**, **C**). The antibodies also labeled spermatogonia (**A**) and spermatocytes (**B**) in the testis but not mature spermatozoa in the vas deferens (**C**). The intensity of labeling in the hypodermis, lateral chords and median chords appeared to decrease towards the posterior end of males in the vicinity of mature sperm (compare **C** to **A** and **B**). Likewise, antibodies also labeled the intestine, hypodermis, lateral chords, and median chords of sexually immature (**D**) and older, mf producing females (**F**). In the older females, intense labeling was seen in the oocytes within the ovaries (**E**) and in early embryos in the uterus (**F**, arrow). Labeling faded in later morulae (**F**, arrowhead) and pretzel stage embryos (**G**) but reappeared on particular regions of the outer surface of curled and stretched microfilariae (**H**, **I**). Abbreviations: cu, cuticle; hy, hypodermis; m, muscle; lc, lateral chords; mc, median chords; i, intestine; ut, uterus; utw, uterine wall; t, testis; vd, vas deferens. Scale bar = 5μm in panel I and 50μm in all other panels. Panels **A**-**G** shows results obtained with the alkaline phosphatase anti-alkaline phosphatase method. Panels **H** and **I** show results obtained with a fluorescein labeled secondary antibody (green); membranes labeled with wheat germ agglutinin appear red, and nuclei stained with DAPI are blue.

**Figure 7:**
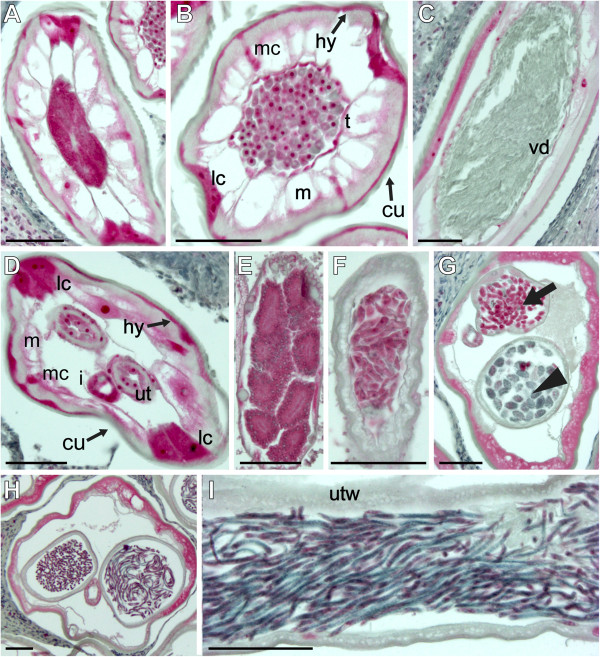
**Localization of the predicted peptide from isotig21532 in *Onchocerca flexuosa *by immunohistochemical labeling.** Polyclonal antibodies raised against a predicted peptide from *O. flexuosa* with sequence similarity to a hypothetical protein from the *Wolbachia* endosymbiont of *Onchocerca volvulus* labeled the hypodermis, lateral, and median chords in the anterior half of male worms (**A**-**C**). Strong labeling was observed in the spermatogonia (**A**) and spermatocytes (**B**) in the testis, but not in mature spermatozoa within the vas deferens (**C**). Sexually immature (**D**) and older, microfilariae producing females both showed antibody labeling in the intestine, hypodermis, lateral chords, and median chords (**G**). In young females, intense labeling was seen on the inner and outer surfaces of the uterus (**D**). In older females, labeling was seen in the ovaries (**E**), oocytes (**F**), and early embryos (**G**, arrow) but not in uterus itself (**G**-**I**). The intensity of labeling decreased dramatically in morula stage embryos (**G**, arrowhead) but increased again in microfilariae (**H**, **I**). Abbreviations: cu, cuticle; hy, hypodermis; m, muscle; lc, lateral chords; mc, median chords; i, intestine; ut, uterus; utw, uterine wall; t, testis; vd, vas deferens. Scale bar = 50μm.

Antibodies against the KLH carrier protein used for antibody production produced no tissue-specific labeling (Figure 5). In contrast, antibodies raised against the HlyD peptide and against the predicted *Wolbachia*-like peptide from isotig21532 both produced tissue-level labeling patterns similar to those seen in the *in situ* hybridization experiments. Antibodies against the HlyD peptide labeled the intestine, hypodermis, lateral chords, and median chords of adult male and female worms (Figure 6A-D and F). They also labeled tissues corresponding to the early stages of sperm development (Figure 6A, B), but did not label mature spermatozoa (Figure 6C). In microfilariae producing females, intense labeling was observed in oocytes (Figure 6E), early embryos (Figure 6F), and regions of microfilariae (Figure 6H, I). Less intense labeling was noted in morula (Figure 6F) and pretzel stages (Figure 6G). The labeling pattern produced by antibodies against the predicted peptide from isotig21532, which shows sequence similarity to a *Wolbachia* hypothetical protein from *O. volvulus*, was nearly identical except that nuclei were more intensely labeled in the stained tissues (Figure 7). With the exception of the male hypodermis, which was not labeled by the RNA probe, it appears likely that these two peptides are mostly retained in the tissues where the corresponding RNAs were synthesized. The similarities between *in situ* hybridization and immunolabeling results make it seem less likely that the multiple bands seen in Western blots probed with these antibodies reflect non-specific labeling or cross-reactivity.

**Figure 8:**
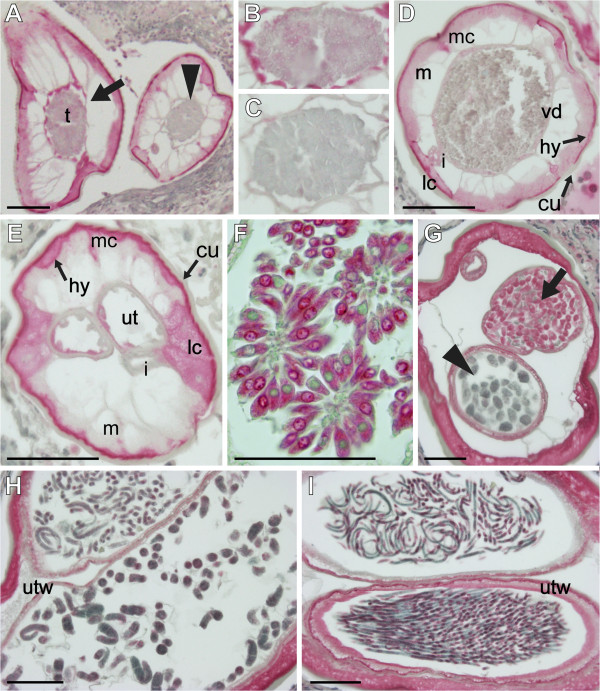
**Localization of the predicted peptide from isotigs12596 in *Onchocerca flexuosa *by immunohistochemical labeling.** Polyclonal antibodies raised against a predicted peptide from *O. flexuosa* with sequence similarity to aminopeptidase P from the *Wolbachia* endosymbiont of *Muscidifurax uniraptor* labeled the intestine, hypodermis, and basal layers of the muscle in male worms (**A**, **D**). Spermatogonia were labeled (see arrow in panel **A**, shown magnified in panel **B**), but spermatocytes (see arrowhead in panel A, shown magnified in panel **C**) and mature spermatozoa were not. The hypodermis was also intensely labeled in sexually immature (**E**) and older, mf producing females (**G**). The older females also showed labeling in the intestine (**G**), oocytes (**F**), uterus branches (**G**, **H**, **I**) and early embryos (**G**, arrow). Lighter labeling was observed in morulae (**G**, arrowhead) and pretzel stage embryos (**H**), but labeling intensity increased in curled and stretched microfilariae (**I**). Abbreviations: cu, cuticle; hy, hypodermis; m, muscle; lc, lateral chords; mc, median chords; i, intestine; ut, uterus; utw, uterine wall; t, testis; vd, vas deferens. Scale bar = 25μm in panel **F** and 50μm in all other panels.

Antibodies against the predicted peptide from isotig12596, a peptide similar to *Wolbachia* aminopeptidase P, produced a labeling pattern somewhat different from the other two antibodies we examined. In this case, strong labeling was seen in the hypodermis in both sexes, but was not as distinct in the lateral chords (Figure 8A, D, E, G). Some labeling was also seen in the basal layers of the muscle in males (Figure 8A, D). We reported similar results for a putative *Wolbachia*-like LolC peptide; while the RNA localization pattern was identical to that seen in Figure 2, immunohistology suggested that the corresponding peptide was restricted to somatic muscles [10]. Again, antibodies against the predicted peptide from isotigs 12596 labeled spermatogonia (Figure 8A, B), but no labeling was detected in spermatocytes (Figure 8A, C), spermatids, or mature spermatozoa (Figure 8D). This could indicate that the corresponding RNA detected in spermatocytes is not translated into protein or that the protein is quickly degraded. The antibody binding patterns seen in female worms and developing offspring were similar to those reported for the other two antibodies (i.e., labeled ovaries, oocytes, early embryos and microfilariae, but not morulae) except that labeling was apparent in the uterine wall of mature females rather than that of immature females (Figure 8E, G, H, I).

It is not surprising that *Wolbachia*-like proteins were detected in tissues or sub-cellular compartments where the corresponding mRNA was not found (e.g., somatic muscles, uterus of mature females, nucleus vs. cytoplasm, etc). Many proteins are produced in a specific tissue (or subset of tissues) but have important functional roles in other regions of the body. In fact, *Wolbachia* gene products are believed to be exported from the endobacteria to interact with host cells, and some of them have been detected among the excretory/secretory products of *Wolbachia*-dependent worms [33].

Different patterns of localization observed for different putative *Wolbachia*-like peptides could indicate that the proteins containing these peptides are responsible for different functions in the worms. Darby et al. [30] proposed a dual role for *Wolbachia* in filarial biology based on their recent genomic analysis of the *Wolbachia* endosymbiont of the cattle parasite *O. ochengi*; they speculated that *Wolbachia* may engage in metabolic provisioning and also serve as a diversion for the vertebrate host immune system. If we hypothesize that *Wolbachia*-like sequences in *O. flexuosa* function as a substitute for the endosymbiont, the localization patterns of proteins with *Wolbachia*-like sequences may provide clues as to which of these two tasks they are most likely to perform. For example, proteins present in somatic muscles and reproductive tissues may be involved in worm metabolism, perhaps granting *O. flexuosa* novel biochemical capabilities compared to other, *Wolbachia*-dependent filarial species (e.g., *de novo* heme, nucleotide or riboflavin synthesis [29,30]). Likewise, proteins with transmembrane domains or secretion peptides that are present in the hypodermis or lateral chords may be released from the worm to distract the host’s immune system by promoting an ineffective Th1 type immune response [30]. Further work will be needed to define the roles of these proteins in the biology of *Wolbachia*-free filarial parasites like *O. flexuosa*.

## Conclusions

The results of this study further support the notion that *Wolbachia-*like sequences are expressed in a *Wolbachia*-free filarial parasite at both the RNA and protein levels; this expression is highly regulated with regard to tissue and parasite stage. Thus far, all of the *Wolbachia*-like RNA probes that have successfully labeled *O. flexuosa* tissues have produced the same labeling pattern: intestine and lateral chords of both sexes, the hypodermis, median chords and uteri of females, and sperm precursors in the male testis. Polyclonal antibodies against predicted *Wolbachia*-like peptides bound to specific bands in Western blots performed with *O. flexuosa* adult worm lysate and to specific tissues in fixed and sectioned parasite specimens. *Wolbachia*-like peptides were sometimes found in the same tissues where the corresponding RNAs were produced and sometimes in other locations. The localization of these proteins within the worm may provide clues regarding their functions by suggesting a role in worm metabolism or in host immune modulation.

## Competing interests

The authors declare that they have no competing interests.

## Authors’ contributions

SM analyzed the *O. flexuosa* transcriptome and proteome, performed bioinformatic analyses and BLAST searches, designed and constructed RNA probes, assisted in the selection of epitopes for antibody production, interpreted results, and drafted the manuscript. KF fixed, embedded and sectioned *O. flexuosa* nodules and performed *in situ* and immunohistochemical labeling studies. KC produced the Western blots. NWB identified, collected and supervised the preservation and preparation of *O. flexuosa* material and edited the manuscript. GW and PF designed and coordinated the study and helped to interpret the results and draft the manuscript. All authors read and approved the final version of the manuscript.

## Supplementary Material

Additional file 1 Table S1. Relative expression of isogroups derived from the *O. flexuosa* transcriptome assembly, reported as sequence reads per kilobase of length. Click here for file

Additional file 2 Table S2. Primers used in the construction of *in situ* hybridization probes. *Wolbachia*-like sequences identified from the transcriptome of *O. flexuosa* were amplified from *O. flexuosa* cDNA. A homolog of the HlyD gene from the *Wolbachia* endosymbiont of *Culex quinquefasciatus* was identified in the *Wolbachia* endosymbiont of *B. malayi.* This sequence was amplified from *B. malayi* genomic DNA which also contains DNA from the *Wolbachia* endosymbiont. (DOCX 41 kb) Click here for file

Additional file 3 Table S3. Target epitopes of anti-peptide antibodies. Epitopes predicted to exhibit favorable immunogenic properties were chosen from regions of *O. flexuosa* peptide translations with sequence similarity to *Wolbachia.* A favorable epitope was also chosen from the HlyD gene from the *Wolbachia* endosymbiont of *Culex quinquefasciatus* (*wCq*). The underlined portion of this epitope matches one of the two peptides identified in our proteomic analysis of *O. flexuosa* worm lysate that mapped to HlyD from *wCq*[10]. (XLSX 39 kb) Click here for file

Additional file 4 Figure S1. Localization of a putative *Wolbachia*-like HlyD transcript in adult *O. flexuosa*. The sense RNA probe (negative control) produced no signal in tissues in either sex (A, C). In female worms, the antisense RNA probe against *Wolbachia* HlyD labeled the hypodermis, lateral chords, median chords, intestine and uterus of young females (B). In male worms, labeling was seen in the lateral and median chords and in spermatocytes within the testis (D) but not in mature spermatozoa in the vas deferens (E). Abbreviations: cu, cuticle; hy, hypodermis; m, muscle; lc, lateral chords; mc, median chords; i, intestine; ut, uterus; t, testis; vd, vas deferens. Scale bar = equals 50μm. (TIFF 9072 kb) Click here for file

Additional file 5 Figure S2. Localization of a transcript with sequence similarity to a *Wolbachia* hypothetical protein in adult *O. flexuosa*. The sense RNA probe (negative control) produced no signal in either sex (A, C). In female worms, the antisense RNA probe against isotigs21532, a sequence similar to that of a hypothetical protein from the *Wolbachia* endosymbiont of *O. volvulus*, showed light labeling of the hypodermis, lateral chords, median chords, intestine and uterus (B). In male worms, labeling was seen in the lateral and median chords and in the germinal zone of the testis (D). Abbreviations: cu, cuticle; hy, hypodermis; m, muscle; lc, lateral chords; mc, median chords; i, intestine; ut, uterus; t, testis. Scale bar = equals 50μm. (TIFF 8374 kb) Click here for file
