# Supplementary material for: Localization of Wolbachia-like gene transcripts and peptides in adult Onchocerca flexuosa worms indicates tissue specific expression
Source: Parasit Vectors. 2013 Jan 2;6:2. doi: 10.1186/1756-3305-6-2 (PMC3549793; doi:10.1186/1756-3305-6-2)
Supplement: Additional file 2 — Table S2. Primers used in the construction of in situ hybridization probes. Wolbachia-like sequences identified from the transcriptome of O. flexuosa were amplified from O. flexuosa cDNA. A homolog of the HlyD gene from the Wolbachia endosymbiont of Culex quinquefasciatus was identified in the Wolbachia endosymbiont of B. malayi. This sequence was amplified from B. malayi genomic DNA which also contains DNA from the Wolbachia endosymbiont. (DOCX 41 kb) [file 1756-3305-6-2-S2.docx]

| Isogroup | Isotig/Locus | Forward Primer (5’-3’) | Reverse Primer (5’-3’) |
| --- | --- | --- | --- |
| isogroup04608 | isotig12596 | tgcttgattctggtggccaat | ctccatgtcctgcaccatgc |
| isogroup13474 | isotig21532 | ggcacgtcctcgttgctgttga | ccaggcggcttaacgaatacttcagg |
| n/a | Wbm0309 | tgtgtcaaaatctcccctaatcg | tggtcggctgtagctccaat |
