# Supplementary figures and images for: Localization of Wolbachia-like gene transcripts and peptides in adult Onchocerca flexuosa worms indicates tissue specific expression
Source: Parasit Vectors. 2013 Jan 2;6:2. doi: 10.1186/1756-3305-6-2 (PMC3549793; doi:10.1186/1756-3305-6-2)

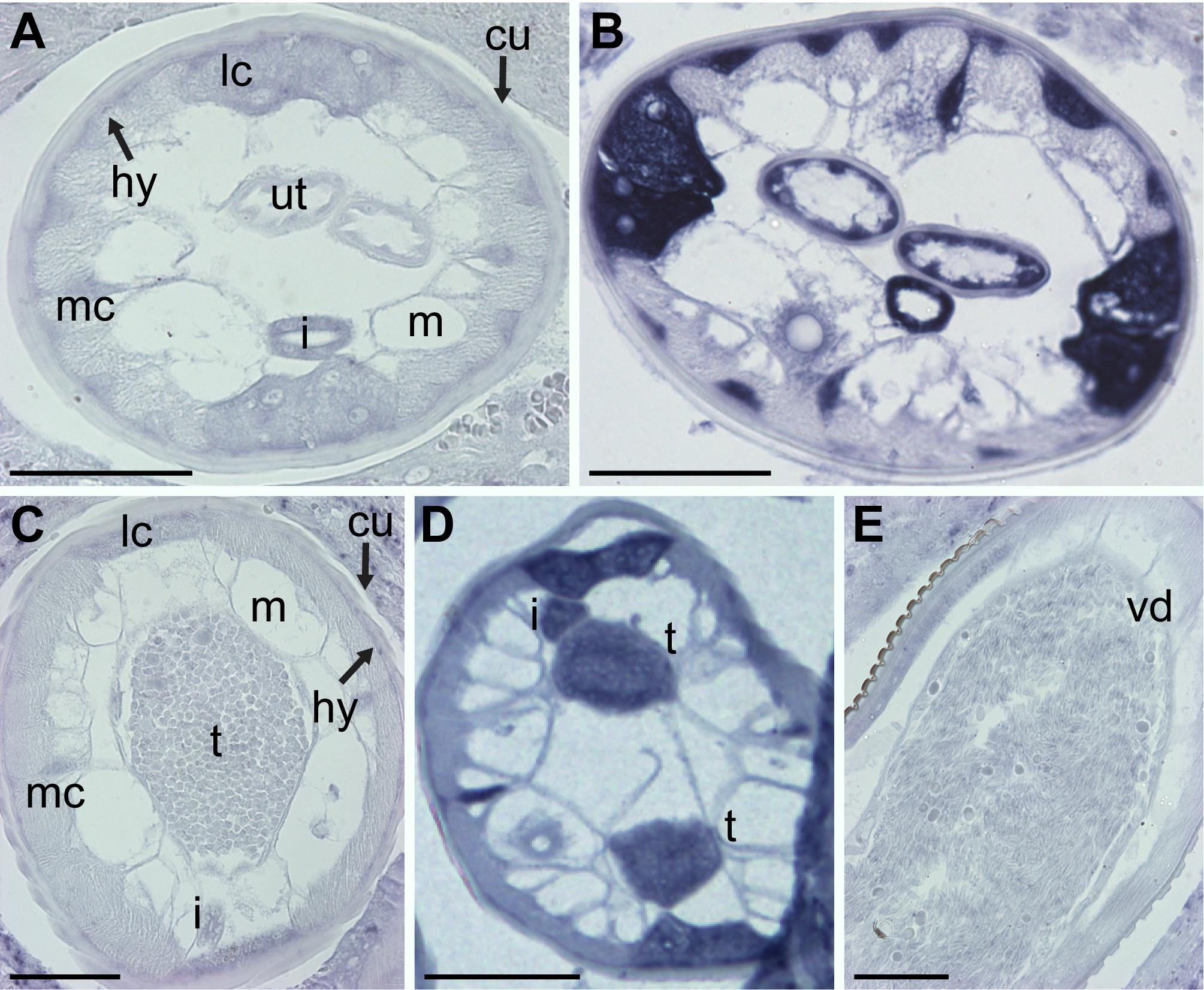

Supplement: Additional file 4 — Figure S1. Localization of a putative Wolbachia-like HlyD transcript in adult O. flexuosa. The sense RNA probe (negative control) produced no signal in tissues in either sex (A, C). In female worms, the antisense RNA probe against Wolbachia HlyD labeled the hypodermis, lateral chords, median chords, intestine and uterus of young females (B). In male worms, labeling was seen in the lateral and median chords and in spermatocytes within the testis (D) but not in mature spermatozoa in the vas deferens (E). Abbreviations: cu, cuticle; hy, hypodermis; m, muscle; lc, lateral chords; mc, median chords; i, intestine; ut, uterus; t, testis; vd, vas deferens. Scale bar = equals 50μm. (TIFF 9072 kb) [file 1756-3305-6-2-S4.tiff]

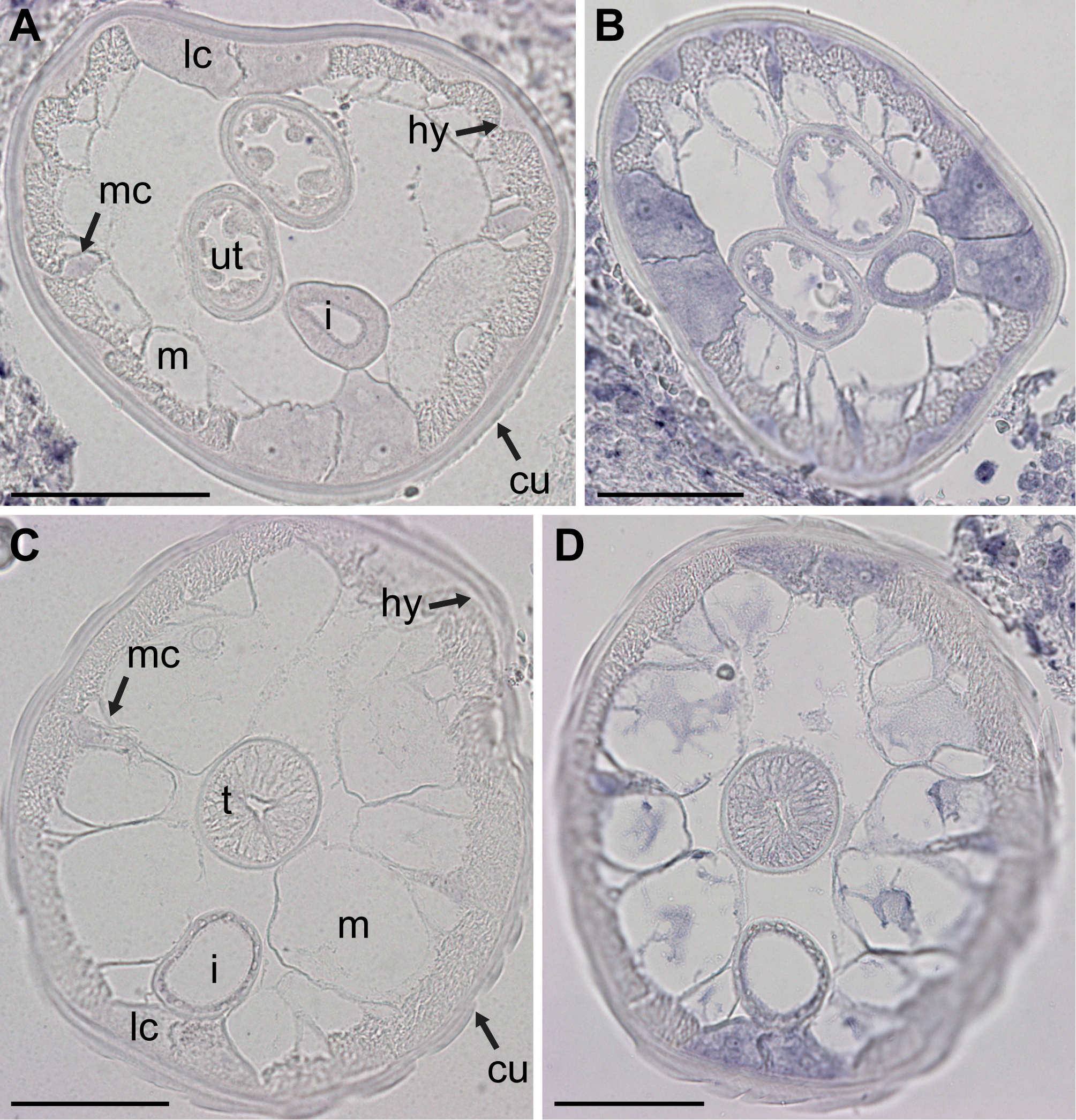

Supplement: Additional file 5 — Figure S2. Localization of a transcript with sequence similarity to a Wolbachia hypothetical protein in adult O. flexuosa. The sense RNA probe (negative control) produced no signal in either sex (A, C). In female worms, the antisense RNA probe against isotigs21532, a sequence similar to that of a hypothetical protein from the Wolbachia endosymbiont of O. volvulus, showed light labeling of the hypodermis, lateral chords, median chords, intestine and uterus (B). In male worms, labeling was seen in the lateral and median chords and in the germinal zone of the testis (D). Abbreviations: cu, cuticle; hy, hypodermis; m, muscle; lc, lateral chords; mc, median chords; i, intestine; ut, uterus; t, testis. Scale bar = equals 50μm. (TIFF 8374 kb) [file 1756-3305-6-2-S5.tiff]
